# Utility of the Respiratory Compensation Point for Estimating Critical Power: Insights From Normoxia and Hypoxia

**DOI:** 10.1002/ejsc.12291

**Published:** 2025-04-03

**Authors:** Simon Marwood, Len Parker Simpson, Daryl P. Wilkerson, Andrew M. Jones, Richie P. Goulding

**Affiliations:** ^1^ School of Health & Sport Sciences Liverpool Hope University Liverpool UK; ^2^ Human Performance Science Research Group University of Edinburgh Edinburgh UK; ^3^ Sport and Health Sciences College of Life and Environmental Sciences St. Luke's Campus University of Exeter Exeter UK; ^4^ Department of Human Movement Sciences Faculty of Behavioral and Human Movement Sciences Amsterdam Movement Sciences Vrije Universiteit Amsterdam Amsterdam the Netherlands

**Keywords:** critical power, hypoxia, respiratory compensation point

## Abstract

We examined the validity of the respiratory compensation point (RCP) in estimating critical power (CP) by determining the relative agreement between them following an acute intervention, hypoxia, which reduces RCP and CP. RCP and CP were determined in normoxia (N: FiO_2_ = 0.21) and hypoxia (H: FiO_2_ = 0.13) with RCP converted to a power output (W) via linear regression of the V̇O_2_–time relationship with correction for the mean response time. RCP and CP were lower in hypoxia compared to normoxia (*p* < 0.001), but there was no difference between CP and RCP in N or H (N: 174 ± 26 (CP) vs. 178 ± 30 (RCP) W; H: 133 ± 19 (CP) vs. 139 ± 22 (RCP) W, *p* = 0.53). In both N (*r* = 0.32, *p* = 0.31) and H (*r* = 0.00, *p* = 0.99), RCP was not correlated with CP. Moreover, the 95% limits of agreement (LOA) were unacceptably wide (N: 3 ± 64 W; H: 7 ± 57 W). There was no correlation between the change in RCP and the change in CP caused by hypoxia (W: *r* = 0.32), with similarly poor 95% LOA (W: −3 ± 62 W). The weak correlations and wide LOA within and between conditions suggest little practical values in using RCP to estimate CP.

**Summary**

- Although both the respiratory compensation point (RCP) and critical power (CP) were similarly reduced in hypoxia compared to normoxia, there was poor agreement between these variables in both conditions. The lack of correlation and wide limits of agreement suggest that RCP is not a reliable proxy for CP.
- The findings support the notion that RCP is affected by a range of physiological mechanisms beyond metabolic acidosis and subsequent carotid body stimulation and thus challenge the assumption that RCP directly reflects the boundary between heavy and severe intensity exercise.

## 1 Introduction

Exercise training is necessary to promote sports performance, and by promoting physical activity and fitness, it is a central aspect of disease prevention and mitigation (Lee et al. 2012). Fundamental to the prescription of exercise training is an understanding of the physiological distinctions between different exercise intensities and how the boundaries between those intensities can be accurately determined. In response to exercise, distinct physiological responses have been identified that exemplify the characteristics of moderate, heavy, severe and extreme intensity exercise, respectively (Whipp and Ward 1982; Poole et al. 1988; Ozyener et al. 2001; Hill et al. 2002; Jones et al. 2008; Vanhatalo et al. 2016). However, the threshold separating heavy and severe intensity exercise is of particular interest because it represents the upper limit beyond which a metabolic steady state is unattainable and oxygen uptake projects inexorably towards maximum values (Poole et al. 2016; Jones et al. 2019), with associated implications for exercise (in)tolerance (Poole et al. 2016; Burnley and Jones 2018; Goulding, Rossiter, et al. 2021).

Unfortunately, the threshold separating heavy and severe intensity exercise cannot be precisely determined from the most commonly applied indices of exercise intensity, such as fractions of maximal heart rate or maximal oxygen uptake (Iannetta, Marinari, et al. 2023). In contrast, when determined with appropriate methods (Jones et al. 2019), critical power (CP), though expressed in units of external performance (i.e., W; or m·s^−1^, N·m for critical speed, torque, depending on the performance metric), reflects an underlying metabolic rate (Barker et al. 2006) that has been demonstrated to demarcate the boundary between heavy and severe exercise (Poole et al. 1988; Hill and Ferguson 1999; Hill et al. 2002; Pringle and Jones 2002; Jones et al. 2008; Burnley et al. 2012; Murgatroyd et al. 2014; Vanhatalo et al. 2016; Black et al. 2017; Lei et al. 2023). However, the determination of CP, which requires multiple maximal exercise trials completed on separate days, is labour‐ and time‐intensive and thus not always practicable. Accordingly, alternative methods to derive the threshold separating heavy and severe intensity exercise have been extensively explored (Burnley et al. 2006; Murgatroyd et al. 2014; Parker Simpson and Kordi 2017; Keir et al. 2018; Goulding, Marwood, et al. 2021; Iannetta, Marinari, et al. 2023).

During ramp incremental exercise, indices of pulmonary gas exchange reveal two distinct thresholds: the gas exchange threshold (GET) and the respiratory compensation point (RCP) (Whipp et al. 1981, 1989). The RCP, typically determined as a metabolic rate (i.e., oxygen uptake), represents the point at which a hyperventilatory response causes the end‐tidal PCO_2_ (P_ET_CO_2_) and arterial PCO_2_ (PaCO_2_) to decline, having previously been stable during a phase known as ‘isocapnic buffering’ (Whipp et al. 1989). The primary mechanisms that bring about respiratory compensation are not clearly established and may be related to muscle afferent feedback or central command (Hagberg et al. 1982; Heigenhauser et al. 1983; Mateika and Duffin 1994; Thornton et al. 2001; Forster et al. 2012). However, a widespread view has been that respiratory compensation occurs in response to the unabated systemic/metabolic acidosis associated with severe intensity exercise, initiated primarily via the stimulation of the carotid artery chemoreceptors, the carotid bodies (Wasserman et al. 1975, 2011; Oren et al. 1982; Rausch et al. 1991). Because a progressive reliance on nonoxidative metabolism, and associated metabolic acidosis, is characteristic of severe intensity exercise (Poole et al. 2016; Jones et al. 2019), this has led some to suggest that the RCP may be a convenient, and valid, surrogate of CP (Keir et al. 2018). However, this contention has proven to be controversial (Keir et al. 2018; Broxterman et al. 2018; Galán‐Rioja et al. 2020).

A robust experimental approach to address the potential validity of proxy measures in establishing accurately the criterion measure is to intervene to acutely alter one variable and observe whether a similar magnitude of change occurs in the other. CP is subject in part to the availability of oxygen, acting either independently (Dekerle et al. 2012; Parker Simpson et al. 2015; Townsend et al. 2017; La Monica et al. 2018; Goulding et al. 2020) or via its role in determining V̇O_2_ kinetics (Vanhatalo et al. 2010; Black et al. 2015; Goulding et al. 2018, 2019; Goulding and Marwood 2023). Hence, if there is a highly conserved, common mechanistic basis relating RCP with CP, the change in CP as a result of exercising in a hyperoxic or hypoxic environment should be matched by a similar change in the RCP. The RCP has been shown to be reduced in hypoxia (Azevedo et al. 2020). However, carotid body chemosensitivity is amplified in hypoxia (Rausch et al. 1991), raising ventilation to the extent that arterial pH is no different, or even higher, during incremental exercise in hypoxia compared to normoxia (Knight et al. 1996; Lovering et al. 2008). The reduction in the RCP in hypoxia (Azevedo et al. 2020) may therefore be augmented relative to the concomitant reduction in CP.

The purpose of the present study was therefore to examine the effect of hypoxia on CP and the RCP. Based on the previous assessments of the agreement between these two variables (Keir et al. 2015; Iannetta, Mackie, et al. 2023), we hypothesised that there would be (i) good agreement between CP and the RCP in normoxia; (ii) poor agreement between the two in hypoxia; and, accordingly, (iii) poor agreement between the change in CP and the change in RCP when moving between the normoxic and hypoxic conditions.

## 2 Materials and Methods

The present study involved a reanalysis of data from a previously published study (Parker Simpson et al. 2015) which had the primary aim of examining the effect of hypoxia on the parameters (i.e., CP and W′) of the power–duration relationship. The present study incorporates the previously published CP and gas exchange threshold data, alongside previously unpublished respiratory compensation point data. For the avoidance of repetition of previously described methods (for which the reader is referred to Parker Simpson et al. (2015)), presented herein is an overview of the procedures most pertinent to the present analysis.

### 2.1 Participants

Thirteen recreationally active females (mean ± SD: age 21 ± 1 year, body mass 69.2 ± 11.9 kg, height 1.66 ± 0.05 m) volunteered and provided written informed consent to participate in this study, which was carried out following the approval from the local research ethics committee. None of the participants reported a history of regular or recent sojourns to altitude. Participants were required to visit the laboratory on 14 occasions over a 4–5 week period irrespective of the menstrual cycle phase (James et al. 2023). A minimum of 24 h separated each visit. Participants were fully familiarised with all testing procedures prior to any experimentation. For each visit, participants were asked to arrive at the laboratory rested (no strenuous exercise performed in the preceding 24 h), fully hydrated, at least 3 h postprandial, and having avoided alcohol and caffeine for the preceding 12 and 6 h, respectively.

### 2.2 Experimental Design

All of the experimental procedures were carried out in a laboratory at sea level. All exercise tests were carried out in both normoxia and hypoxia, with the hypoxic environment induced via the inspiration of gas, with an average O_2_ fraction of 0.128 ± 0.02, from a 1000 L Douglas bag. Participants were blinded to the condition via a concealed 3‐way value, where inspired gas was taken either from the 1000‐L Douglas bag or room air. Each exercise test preceded by 5 min of ‘unloaded’ cycling (20 W) while inhaling the given inspirate for the test in order to equilibrate the body O_2_ stores.

In a randomised order, participants initially completed in each condition a ramp incremental test (25 W·min^−1^) to the limit of tolerance. Thereafter, participants completed five constant power tests to the limit of tolerance in each condition in a randomised order (with respect to condition and power). The required power for these trials was chosen to result in a limit of tolerance ranging between 2 and 15 min. For both incremental and constant power exercise tests, participants were instructed to maintain their preferred cadence throughout (± 5 rpm), with the limit of tolerance defined as the time at which cadence fell by > 10 rpm for more than 5 s. Participants were asked to remain seated on the ergometer, and strong verbal encouragement was provided by the experimenter.

### 2.3 Data Analysis

Pulmonary gas exchange data were averaged into 10 s bins. Peak V̇O_2_ (V̇O_2_peak) was defined as the highest 30 s rolling average during the incremental exercise test. The RCP was determined via the visual inspection of the V̇O_2_ at which the end‐tidal pressure of CO_2_ (PETCO_2_) began to fall after a period of isocapnic buffering (i.e., stable PETCO_2_), corroborated by a sharp increase in ventilation (V̇_E_) with respect to V̇CO_2_ (i.e., a breakpoint in the V̇_E_/V̇CO_2_ response) (Whipp et al. 1989). The GET was determined via the V‐slope method (Beaver et al. 1986), verified by observing the V̇O_2_ at which there is a sustained increase in V̇_E_/V̇O_2_ with no change in V̇_E_/V̇CO_2_ and an increase in PETO_2_ without a concomitant rise in PETCO_2_. V̇_E_ at GET (V̇_EGET_) and RCP (V̇_ERCP_) were determined via the linear regression of the V̇_E_ response between the time at which GET and RCP were expressed.

RCP and GET were converted to a power by correcting downward the instantaneous power with respect to the response time of V̇O_2_ (i.e., mean response time, MRT) during the ramp incremental exercise test. The MRT was estimated by observing the time at which a backwards extrapolation of the linear portion of the V̇O_2_–power relationship was equal to the value of V̇O_2_ observed during baseline pedalling at 20W (Boone and Bourgois 2012).

CP (and W′) was determined from the power–duration relationship derived from the constant power exercise tests. Linear regression was used to provide two sets of CP and *W*′ estimates from the results of the prediction trials, using the work time (*W* = CP·*t* + *W*′) and the 1/time (*P* = *W*′·(1/*t*) + CP) models. For each individual case, the model providing the lowest standard errors and the highest *r* ^2^ was chosen to provide the CP and *W*′ parameter estimates. In the present analysis, once *W*′ was determined in order to also derive CP, *W*′ was not considered further.

### 2.4 Statistics

The effects of the condition (hypoxia vs. normoxia) on V̇O_2_peak, RCP (%V̇O_2_peak), GET, V̇_E_peak, V̇_ERCP_ and V̇_EGET_ were analysed using paired sample *t*‐tests. RCP and CP were analysed by a 2‐way repeated measures ANOVA for conditions (normoxia vs. hypoxia) and variables (RCP vs. CP). Post hoc analysis for these analyses was via a simple effects analysis with Bonferroni correction applied. Violations for sphericity were corrected by Greenhouse‐Geisser (GG) where the GG Epsilon < 0.75, and corrected by Hyunh‐Feldt where the GG Epsilon was > 0.75. Relationships between variables were analysed via a Pearson correlation; agreement between variables was analysed via average bias and 95% limits of agreement (LOA). Data are presented as mean ± standard deviation with the statistical significance set at *p* < 0.05. Statistical analysis was undertaken using IBM SPSS statistics version 29.0.1.0.

## 3 Results

**FIGURE 1:**
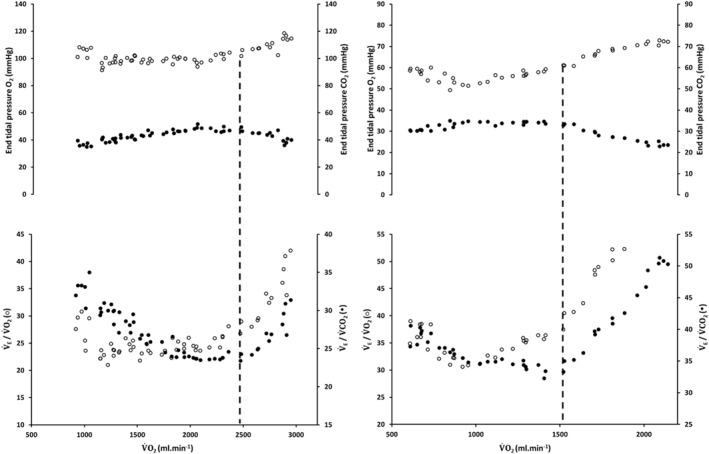
Representative example of RCP determination in normoxia (left‐hand panel) and hypoxia (right‐hand panel). Open‐circles: end‐tidal pressure and ventilatory equivalent for O_2_; closed‐circles: end‐tidal pressure and ventilatory equivalent for CO_2_.

Due to one file being corrupted, data are presented for 12 females (age: 21 ± 1 year, height: 1.7 ± 0.1 m; mass: 70 ± 12 kg). Exercise tolerance in the constant power tests was 180 ± 18 s to 790 ± 143 s (normoxia) and 185 ± 11 s to 739 ± 184 s (hypoxia). Table 1 shows ramp incremental exercise test outcomes and CP; the V̇O_2_peak was lower in hypoxia compared to normoxia (*p* < 0.01) but was not different within conditions between incremental and constant power exercise tests (data not shown; see Parker Simpson et al. (2015)). GET was lower in hypoxia compared to normoxia whether expressed as an oxygen uptake (*p* < 0.001) or power (*p* < 0.001). However, GET was not different between conditions when expressed as % V̇O_2_peak (*p* = 0.13) or %RCP (*p* = 0.73). RCP (%V̇O_2_peak) was also not different between conditions (*p* = 0.55). V̇_E_peak (*p* = 0.60) and V̇_ERCP_ (*p* = 0.72) were not different between conditions; however, V̇_EGET_ was lower in normoxia compared to hypoxia (*p* = 0.04). Representative plots of RCP determination in normoxia and hypoxia can be seen in Figure 1.

**TABLE 1:** Outcomes from ramp incremental exercise and critical power.

	Normoxia	Hypoxia	Δ%
Ramp incremental exercise			
V̇O_2_peak (mL·min^−1^)	2911 ± 440	2354 ± 278*	−19
RCP (mL·min^−1^)	2260 ± 405	1856 ± 242*	−18
RCP (W)	178 ± 30	139 ± 22*	−22
RCP (%V̇O_2_peak)	77.5 ± 7.6	79.0 ± 7.5	1.9
GET (mL·min^−1^)	1479 ± 203	1247 ± 182*	−16
GET (W)	90 ± 18	66 ± 13*	−27
GET (%V̇O_2_peak)	51.2 ± 3.9	53.2 ± 5.6	4.0
GET (%RCP)	66.6 ± 7.3	67.6 ± 7.1	1.6
V̇_E_peak (L·min^−1^)	128 ± 13	126 ± 13	−1
V̇_ERCP_ (L·min^−1^)	69 ± 15	70 ± 12	9
V̇_EGET_ (L·min^−1^)	36.4 ± 5.5	39.7 ± 7.0*	1.9
Critical power (W)	174 ± 26	133 ± 19*	−24

*Note:* Data are mean ± SD. V̇O_2_peak, highest rolling 30 s average during incremental exercise.

Abbreviations: CP, critical power; GET, gas exchange threshold; RCP, respiratory compensation point; V̇_E_peak, highest rolling 30 s average during incremental exercise; V̇_ERCP_ and V̇_EGET_, ventilation at RCP and GET, respectively.

* *p* < 0.01 versus normoxia.

### 3.1 RCP Versus CP

**FIGURE 2:**
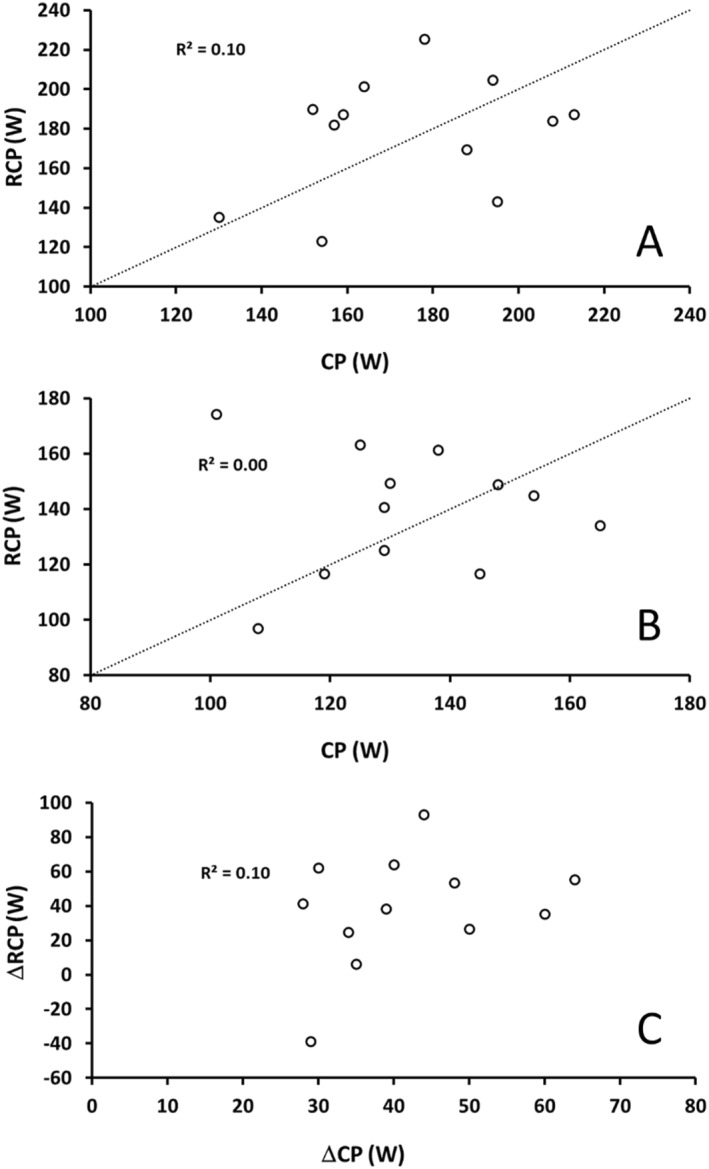
Correlation between critical power (CP) and the respiratory compensation point (RCP) (panel (A), normoxia; panel (B), hypoxia) and the difference (Δ) between conditions (C). Dashed lines reflect the line of identity (removed for clarity on panel (C)). Explained variance (*R* ^2^) displayed on the inset of each panel.

RCP and CP were lower in hypoxia compared to normoxia (main effect condition, *p* < 0.001), with no difference between RCP and CP (main effect variable, *p* = 0.53, interaction condition × variable, *p* = 0.71) (Table 1). RCP was not correlated with CP either in normoxia or hypoxia (normoxia: *r* = 0.32, *p* = 0.31; hypoxia: *r* = 0.00, *p* = 0.99) (Figure 2A,B). The reduction (Δ) in RCP with hypoxia was also not correlated with ΔCP (*r* = 0.32, *p* = 0.31; Figure 2C).

**FIGURE 3:**
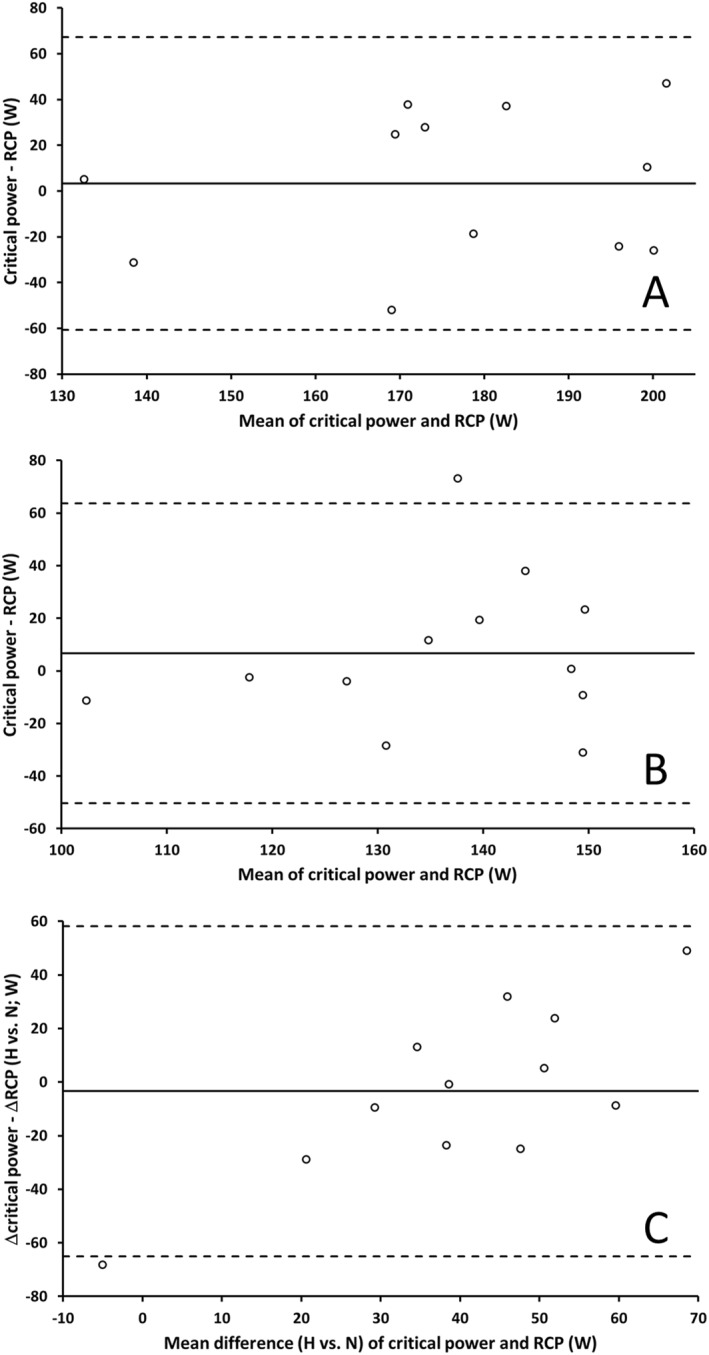
Ninety‐five percent limits of agreement (LOA) between critical power (CP) and the respiratory compensation point (RCP) (panel (A), normoxia; panel (B), hypoxia) and the difference (Δ) between conditions (C). Solid line illustrates the mean bias, and dashed lines illustrate the 95% LOA.

In normoxia, the mean difference and 95% LOA between RCP and CP were 3 ± 64 W (−61, +67 W) (Figure 3A). In hypoxia, the mean difference and 95% LOA between RCP and CP were 7 ± 57 W (−50, +64 W) (Figure 3B). The mean difference and 95% LOA between ΔRCP and ΔCP were −3 ± 62 W (−65, +58 W) (Figure 3C).

## 4 Discussion

The results of the present study show that while there was no systematic difference between the RCP and CP in hypoxia and normoxia, there was poor agreement between these variables in both conditions. RCP was not correlated with CP in either normoxia or hypoxia, with unacceptably wide 95% LOA (± 57–64 W) and similarly poor agreements were observed when examining the changes (Δ) in RCP and CP in response to hypoxia. There was no correlation between ΔRCP and ΔCP, with similarly wide 95% limits of agreement as for the absolute comparisons. The results therefore question the notion that the RCP and CP share a common mechanistic basis and suggest that there is little practical values in using RCP to estimate CP, and thus the heavy‐severe threshold.

The traditional interpretation of the RCP phenomenon is that it is initiated primarily via the stimulation of the carotid bodies (Wasserman et al. 1975; Oren et al. 1982; Rausch et al. 1991) arising from excessive acidosis following the failure of the bicarbonate buffer system to halt the inexorable increase in arterial [H^+^] during incremental exercise (Keir et al. 2022). According to this interpretation, the notion that the RCP reflects the threshold separating heavy and severe intensity exercise follows from the understanding that severe intensity exercise is characterised by (i) the inability to attain a metabolic steady state; (ii) progressive reliance on nonoxidative metabolism; and (iii) consequent unabated metabolic acidosis (Whipp and Ward 1982). The significant attention that RCP has recently received also arises because of its relative ease of measurement during a single incremental exercise test while utilising standard pulmonary gas exchange practices (Dekerle et al. 2003; Keir et al. 2015, 2022; Caen et al. 2018, 2022; Galán‐Rioja et al. 2020; Goulding, Marwood, et al. 2021; Tiller et al. 2023). Since CP is a validated index of this threshold when measured appropriately (Hill and Ferguson 1999; Hill et al. 2002; Pringle and Jones 2002; Jones et al. 2008, 2019; Burnley et al. 2012; Murgatroyd et al. 2014; Vanhatalo et al. 2016; Poole et al. 2016; Black et al. 2017; Lei et al. 2023), the agreement, or lack thereof, between RCP and CP has been the subject of scrutiny. The outcome of such studies appears equivocal, with some finding poor agreement between RCP and CP (Broxterman, Ade, Craig, et al. 2015; Leo et al. 2017; Caen et al. 2018, 2022; Tiller et al. 2023), yet others reporting remarkable agreement (Keir et al. 2015; Iannetta, Mackie, et al. 2023). A more robust way to examine the validity of RCP in estimating CP is to intervene to alter CP and observe the effects on RCP. Relatively few studies have taken such an approach (Broxterman, Ade, Barker, et al. 2015; Caen et al. 2018). Accordingly, the present study sought to examine the effects of an acute intervention, hypoxia, known to bring about a reduction in CP (Parker Simpson et al. 2015).

The RCP might be predicted to be reduced in hypoxia, compared to normoxia, because the resulting reduction in intracellular oxygen tension mandates a greater disruption to metabolic stability (Haseler et al. 1998, 1999; Hogan et al. 1999) and a resultant increase in cellular acidosis during incremental exercise (Richardson et al. 1998; Hogan et al. 1999). Indeed, the RCP has previously been shown to be reduced by hypoxia (Azevedo et al. 2020). Similarly, CP is dependent on oxygen availability, reflecting both changes to metabolic stability and its role in determining oxygen uptake kinetics (Poole et al. 2016; Goulding and Marwood 2023). It is perhaps therefore not surprising that these variables were similarly reduced in hypoxia compared to normoxia (Table 1, Figure 3). Rather, the outstanding feature of the present analysis is the marked lack of agreement between RCP and CP, both in normoxia and hypoxia, and when considered as the change between conditions.

That the RCP is a function of excessive acidosis and carotid body chemosensitivity is supported by studies demonstrating its suppression in patients who have undergone carotid body resection (Wasserman et al. 1975) and faster ventilatory kinetics following pharmacologically induced metabolic acidosis (Oren et al. 1982). However, in a number of contexts, including glycogen depletion, prior exercise, McArdle's disease, exercise‐induced muscle damage, alkalosis and differences in ramp rate, respiratory compensation and metabolic acidosis have been dissociated (Hagberg et al. 1982; Heigenhauser et al. 1983; Mateika and Duffin 1994; Scheuermann and Kowalchuk 1998; Ozcelik et al. 1999; Meyer et al. 2004; Davies et al. 2011). Moreover, during ramp exercise, the RCP occurs at a higher metabolic rate than the GET, following a period of ‘isocapnic buffering’ (Whipp et al. 1989). In contrast, during slowly incremented (particularly ‘step’) exercise, there is a coincidence of respiratory compensation with the gas exchange threshold (Wasserman et al. 1973; Wasserman and Whipp 1975). Therefore a fundamental issue for the use of RCP as a proxy for the heavy‐severe threshold is its protocol dependency, extending to it being undetectable in certain conditions (Wasserman and Whipp 1975; Scheuermann and Kowalchuk 1998; Tiller et al. 2023).

The role of the incrementation rate in the appearance of the RCP seemingly reflects the carotid bodies having (unknown) time delays or [H^+^] thresholds with respect to the intracellular expression of the systemic acidosis (Buckler et al. 1991). Yet, a viable mechanistic link between the RCP and the upper limit of the metabolic steady state on the basis of acidosis necessitates a prerequisite matching of the time course and magnitude of [H^+^] efflux from the muscle cell, transporting through the circulation and interaction with the carotid body cell characteristics. However, individual differences in ventilatory chemosensitivity are significant contributors to the RCP (Takano 2000). Furthermore, arterial [H^+^] has been shown to be essentially unchanged in hypoxia compared to normoxia during incremental exercise (Knight et al. 1996; Lovering et al. 2008), suggesting ventilatory adjustments can occur independently of, and more rapidly than can be ascribed to, systemic perturbations that follow from increases in metabolic instability (Rausch et al. 1991). Hence, whether the RCP arises due to progressive or reflexive ventilatory kinetics, any alignment with the heavy‐severe domain boundary may be coincidental rather than causal, with the present poor agreement between RCP and CP therefore unsurprising. Taken together, in accordance with previous commentaries on this matter (Nicolò et al. 2020), the poor inter‐individual agreement between RCP and CP suggests other inputs to the RCP phenomenon that are distinct to intramuscular metabolic (in)stability and are more important, such as central command and muscle afferent feedback. In turn, the stimulation of ventilation via central command and/or muscle afferent feedback may be secondary to a wide range of factors, including muscle fibre recruitment and disruption, microvascular distension, nociception (Heigenhauser et al. 1983; Haouzi et al. 2004; Davies et al. 2011; Dempsey et al. 2014) and blood O_2_ tension (Azevedo et al. 2020, present study).

A consideration for the determination of the RCP in hypoxia is the changes to ventilation that ensue. Acute hypoxia, as under examination herein, results in augmented ventilation that is subsequently suppressed (though remaining above the normoxic baseline) within a timescale of 20–25 min, at least at rest (Easton et al. 1988). Whether the timescale of hypoxic exposure affects the ventilatory response during exercise is not known; however, ventilation has been shown to be higher at a given absolute exercise intensity throughout incremental exercise in hypoxia (Mekjavic et al. 1987). This was also demonstrated in the present study by the similar ventilation at the GET, RCP and peak incremental power between conditions, despite the absolute power at these instances being lower in hypoxia (Table 1). Notably, the increase in ventilation induced by hypoxia is driven primarily by an increase in the tidal volume rather than breathing frequency (Mekjavic et al. 1987). Since the RCP appears to be a phenomenon related to breathing frequency (Nicolò et al. 2020), this should serve to protect the integrity of the RCP measurement and avoid a ‘pseudo’ RCP, as can happen with prior hyperventilation for the GET (Ozcelik et al. 1999).

When comparing the RCP and CP, a primary methodological issue arises in that the former is expressed as an oxygen uptake and the latter as a power output. Hence, any comparison necessitates a conversion from one unit to the other. Ideally, any such approach would include confirmatory bouts of exercise at a fixed power output to ensure a match with oxygen uptake, and vice versa. This of course would be highly labour‐intensive, and for the present analysis, such data were not available. Hence, we estimated the power output at RCP by correcting for the MRT of V̇O_2_ that accounts for the lag in pulmonary V̇O_2_ relative to power output during ramp incremental exercise (Whipp et al. 1981).

In estimating the power at the RCP, it was necessary to utilise a standardised approach for defining the MRT. We calculated MRT as the time at which extrapolated baseline and ramp V̇O_2_ data intersected (Boone and Bourgois 2012). The original study from which the present data were drawn did not undertake a pre‐ or post‐ramp constant power bout of exercise; hence, we were unable to employ the methods suggested by Iannetta et al. (2020), which were reported to enhance the consistency of MRT derivation (Iannetta et al. 2019). Accordingly, there is an inherent, but unknown, degree of error when converting between V̇O_2_ and power output that will contribute to the poor agreement between the RCP and CP we have established herein, and that may have been improved had we been able to utilise the methods of Iannetta et al. (2020). However, the extent of the disagreement, with 95% LOA of ∼40% (W) of the group mean value, strongly suggests that the underlying agreement between RCP and CP is weak or indeed absent. Indeed, there was no correlations between the RCP and CP in either condition and no correlation between the change in RCP and CP between conditions.

In conclusion, we used hypoxia as an intervention to bring about an acute alteration to CP and to observe the effects on RCP. Hypoxia reduced both CP and RCP to a similar extent, with no difference between them. However, there was an unacceptably poor agreement between these variables both in absolute terms and when considered as the change between conditions. We propose that the multitude of factors contributing to the RCP, many of which exist independently of muscle metabolism, preclude it from providing a robust estimate of CP, and thus the heavy‐severe threshold.

## Ethics Statement

The data presented herein were collected following research ethics approval from the local ethics committee at the University of Exeter.

## Conflicts of Interest

The authors declare no conflicts of interest.

## Data Availability

Data are available on request from the corresponding author.
